# Structure of aldose reductase from *Giardia lamblia*

**DOI:** 10.1107/S1744309111030879

**Published:** 2011-08-16

**Authors:** M. Ferrell, J. Abendroth, Y. Zhang, B. Sankaran, T. E. Edwards, B. L. Staker, W. C. Van Voorhis, L. J. Stewart, P. J. Myler

**Affiliations:** a Seattle Structural Genomics Center for Infectious Disease, USA; b Seattle Biomedical Research Institute, 307 Westlake Avenue North, Suite 500, Seattle, WA 98109, USA; c Emerald BioStructures Inc., 7869 NE Day Road West, Bainbridge Island, WA 98110, USA; d Berkeley Center For Structural Biology, Ernest Orlando Lawrence Berkeley National Laboratory, 1 Cyclotron Road, Berkeley, CA 94720, USA; e School of Medicine, University of Washington, Seattle, WA 98195, USA

**Keywords:** aldose reductases, *Giardia lamblia*, SSGCID

## Abstract

*Giardia lamblia* is an anaerobic aerotolerant eukaryotic parasite of the intestines. It is believed to have diverged early from eukarya during evolution and is thus lacking in many of the typical eukaryotic organelles and biochemical pathways. Most conspicuously, mitochondria and the associated machinery of oxidative phosphorylation are absent; instead, energy is derived from substrate-level phosphorylation. Here, the 1.75 Å resolution crystal structure of *G. lamblia* aldose reductase heterologously expressed in *Escherichia coli* is reported. As in other oxidoreductases, *G. lamblia* aldose reductase adopts a TIM-barrel conformation with the NADP^+^-binding site located within the eight β-strands of the interior.

## Abstract 2

The 1.75 Å resolution crystal structure of aldose reductase from *G. lamblia*, the etiological agent of giardiasis, is reported.

## 1. Introduction

### 1.1. *Giardia lamblia*

*G. lamblia* is the etiological agent of giardiasis, a common cause of diarrheal disease in the developing world. It is an anaerobic aerotolerant eukaryotic parasite of the lower intestine. *Giardia* sp. are believed to be basal eukaryotes, with a minimized genome and simplified metabolic pathways (Morrison *et al.*, 2007 ▶). Mitochondria and the machinery of oxidative phosphorylation are absent, with energy being derived from substrate-level phosphorylation. Carbohydrate metabolism is largely limited to d-glucose (Lindmark, 1980 ▶), while other sugars, sugar alcohols, Krebs-cycle intermediates and organic acids have not been found to be utilized. Glucose is oxidized to ethanol and acetate as the fermentation end products (Lindmark, 1980 ▶; Jarroll *et al.*, 1981 ▶; Schofield *et al.*, 1991 ▶), with ethanol being the dominant product under anaerobic conditions and acetate predominating under aerobic conditions. When cultured in glucose-depleted media, *Giardia* grow slowly, achieve lower cell densities and secrete little ethanol (Schofield *et al.*, 1990 ▶). While glucose appears to be the preferred carbon source, it is not however essential for *Giardia* growth. In the absence of glucose the carbon skeletons of amino acids are used as a substitute, with alanine secreted as the waste product. Ethanol production is largely absent under glucose-limited conditions. Alcohol dehydrogenase (ADH), while present in *Giardia*, does not appear to play a role in the production of the ethanol waste product. Cultures grown in the presence of pyrazole, an inhibitor of ADH, show no reduction in ethanol production. Instead, it is believed that ethanol is produced by the reduction of acetaldehyde by aldose reductase in an NADPH-dependent reaction. *Giardia* grown in the presence of sodium valproate, an inhibitor of aldose reductase, show diminished ethanol production and limited growth (Schofield *et al.*, 1991 ▶).

### 1.2. Aldose reductase

Aldose reductases (EC 1.1.1.21) are ubiquitous NADPH-dependent oxidoreductases that catalyze the reduction of aldehydes to alcohols. As the name suggests, the aldehyde substrates have been classically characterized as aldose sugars; however, in recent years there has been an increased appreciation of the potential of these enzymes to detoxify small aldehydes. Typical aldose reductases exhibit the TIM-barrel fold with eight α-helices on the exterior and an eight-stranded β-barrel in the interior. Binding of the NADPH cofactor is facilitated by a deep elliptical pocket near the C-terminal end of the β-barrel (Borhani *et al.*, 1992 ▶; Wilson *et al.*, 1992 ▶; Rondeau *et al.*, 1992 ▶). Mutagenic studies have shown that tight cofactor binding is dependent on a conserved arginine residue (Arg269 in PDB entry 3kbr; Bohren *et al.*, 2005 ▶). The active site consists of two distinct pockets: a rigid anion-binding pocket and a flexible hydrophobic specificity pocket. A third region near the C-terminus has been shown to change position upon binding certain ligands (Urzhumtsev *et al.*, 1997 ▶; Kinoshita *et al.*, 2002 ▶; Podjarny *et al.*, 2004 ▶). Aldose reductase is common to both prokaryotes and eukaryotes, with TIM-barrel folds being reported from a diversity of species. Aldose reductase functions by a sequential ordered mechanism (Kubiseski *et al.*, 1992 ▶; Grimshaw *et al.*, 1995 ▶), with NADPH being bound before substrate. Activity involves a stereospecific transfer of the 4-pro-*R* hydride from NADPH to the substrate carbonyl C atom followed by the proton­ation of the substrate carbonyl O atom by a conserved tyrosine residue (Tyr40 in 3kbr). Proton transfer in the active site is assisted by a hydrogen bond between the active tyrosine and the ∊-amino group of a conserved lysine (Lys71 in 3kbr), which is itself linked by a salt bridge to a conserved aspartic acid (Asp35 in 3kbr). The substrate is oriented in the active site by a conserved histidine residue (His104 in 3kbr) (Del Corso *et al.*, 2008 ▶).

Owing to its role in human disease, human aldose reductase has been the subject of over thirty years of intense research. In diabetic hyperglycemia, the hexokinase of insulin-independent tissues becomes saturated with glucose, causing excess glucose to instead be shunted into the polyol pathway, where aldose reductase converts it to sorbitol. Disease results from sorbitol-induced hyperosmotic swelling and oxidative stress owing to the lowered glutathione concentration caused by the depletion of cellular NADPH reserves. A large number of inhibitors have been identified that bind the active site and many crystal structures of mammalian aldose reductases with bound inhibitors have been solved. Unfortunately, despite these efforts, very few of these compounds have made it past clinical trials (Del Corso *et al.*, 2008 ▶).

## 2. Methods

### 2.1. Protein expression and purification

Full-length aldose reductase (Morrison *et al.*, 2007 ▶) from *G. lamblia* ATCC 50803 was cloned into pAVA0421 vector (Alexandrov *et al.*, 2004 ▶) by ligand-independent cloning (LIC; Aslanidis & de Jong, 1990 ▶) to produce a construct with an N-terminal hexahistidine tag that is cleavable with 3C protease (the entire tag sequence was MAHHHHHHMGTLEAQTQ′GPGS-ORF, in which the 3C cleavage site is marked by a prime). Protein was expressed in *Escherichia coli* BL21 (DE3) cells in 2 l auto-induction medium (Studier, 2005 ▶) in a LEX bioreactor (Harbinger, Markham, Ontario, Canada) at 293 K for 72 h, after which the harvested cells were flash-frozen in liquid nitrogen. The frozen cell pellet was thawed and resuspended by vortexing in 200 ml lysis buffer [20 m*M* HEPES pH 7.4, 300 m*M* NaCl, 5% glycerol, 30 m*M* imidazole, 0.5% CHAPS, 10 m*M* MgCl_2_, 3 m*M* β-mercaptoethanol, 1.3 mg ml^−1^ protease-inhibitor cocktail (Roche, Basel, Switzerland) and 0.05 mg ml^−1^ lysozyme]. The cell suspension was packed on ice and disrupted by sonication for 15 min in 5 s pulses at 70% amplitude using a Branson 450D Sonifier (Branson Ultrasonics, Danbury, Connecticut, USA). The lysate was incubated with 20 µl Benzonase nuclease (EMD Chemicals, Gibbstown, New Jersey, USA) for 40 min at room temperature under gentle agitation. The lysate was clarified by centrifugation with a Sorvall RC5 at 10 000 rev min^−1^ for 60 min at 277 K in a F14S rotor (Thermo Fisher, Waltham, Massachusetts, USA). The clarified solution was syringe-filtered through a 0.45 µm cellulose acetate filter (Corning Life Sciences, Lowell, Massachusetts, USA). The tagged protein was purified by affinity chromatography using a HisTrap FF 5 ml column (GE Biosciences, Piscataway, New Jersey, USA) equilibrated in binding buffer (25 m*M* HEPES pH 7.0, 300 m*M* NaCl, 5% glycerol, 30 m*M* imidazole, 1 m*M* DTT) and eluted with 500 m*M* imidazole in the same buffer. To cleave the N-terminal affinity tag, peak fractions were pooled and assayed for concentration by 280 nm spectrophotometry; 3C protease (Alexandrov *et al.*, 2001 ▶) was mixed with the target in a 1:50 ratio and dialyzed overnight at 277 K against cleavage buffer (20 m*M* HEPES pH 7.6, 200 m*M* NaCl, 5% glycerol, 1 m*M* DTT). Uncleaved target, protease and cleaved tag were removed by a second round of affinity chromatography using a 5 ml HisTrap column. The flowthrough and wash from secondary affinity chromatography were pooled and concentrated using an Amicon Ultra-15 30 kDa molecular-weight cutoff concentrator (Millipore, Billerica, Massachusetts, USA). The concentrated sample was further purified by size-exclusion chromatography (SEC) using a Superdex 75 26/60 column (GE Biosciences) equilibrated in SEC buffer (20 m*M* HEPES pH 7.0, 300 m*M* NaCl, 5% glycerol and 2 m*M* DTT) attached to an ÄKTAprime plus FPLC system (GE Biosciences). Peak fractions were collected and assessed for purity by SDS–PAGE on 4–12% NuPAGE gels (Invitrogen, Carlsbad, California, USA) with Coomassie staining using SimplyBlue Safestain (Invitrogen). Pure fractions were pooled, concentrated to 23 mg ml^−1^ and flash-frozen in liquid nitrogen. The final concentration was determined by 280 nm spectrophotometry and the final purity was assayed by SDS–PAGE.

### 2.2. Crystallization

With the purified protein at a concentration of 25.2 mg ml^−1^ in SEC buffer, two sparse-matrix screens were set up using JCSG+ (Emerald BioSystems, Bainbridge Island, Washington, USA), PACT (Molecular Dimensions, Suffolk, England), Index and Crystal Screen (Hampton Research, Aliso Viejo, California, USA) following an extended Newman’s strategy (Newman *et al.*, 2005 ▶). 0.4 µl protein solution was mixed with 0.4 µl well solution and equilibrated against 100 µl reservoir using 96-well Compact Jr crystallization plates from Emerald BioSystems. Crystals were found under several PEG con­ditions, while crystals suitable for X-ray diffraction experiments were obtained from PACT condition H6: 20% PEG 3350, 200 m*M* sodium formate, 100 m*M* Bis-Tris propane. The crystals were cryoprotected by soaking them in a buffer consisting of 25% ethylene glycol mixed with reservoir solution. The crystals were vitrified by plunging them directly into liquid nitrogen.

### 2.3. Data collection and structural determination

Diffraction data were collected on the Berkeley Center for Structural Biology ALS 5.0.1 beamline as part of the Collaborative Crystallography program. The beamline uses a wavelength of 0.9774 Å and is equipped with an ADSC Quantum 210 CCD detector. The data were reduced in the monoclinic space group *C*2 to 1.75 Å resolution with *XDS*/*XSCALE* (Kabsch, 1988 ▶, 2010 ▶; see Table 1 ▶).

Packing density (Matthews, 1968 ▶) suggested the presence of two molecules of aldose reductase in the asymmetric unit (*V*_M_ = 1.58 Å^3^ Da^−1^, 52% solvent). A search of the PDB for sequence homology yielded a human aldose-reductase-like protein (AKR1B10) as the closest homolog with known structure (PDB entry 1zua; Gallego *et al.*, 2007 ▶), with 43% sequence identity. A search model was generated from molecule *A* of PDB entry 1zua using the *CCP*4 program *CHAINSAW* (Stein, 2008 ▶; Winn *et al.*, 2011 ▶). The structure was solved by molecular replacement with the *CCP*4 program *Phaser* (McCoy *et al.*, 2007 ▶); two molecules could be placed with high *Z* scores. The model was then iteratively extended manually using *Coot* (Emsley & Cowtan, 2004 ▶) followed by cycles of reciprocal-space refinement with the *CCP*4 program *REFMAC*5 (Murshudov *et al.*, 2011 ▶). The final model was validated with the validation tools within *Coot* and with *MolProbity* (Chen *et al.*, 2010 ▶). 

The final model was refined at 1.75 Å resolution to *R*_work_ = 0.144 and *R*_free_ = 0.173 with good stereochemistry (see Table 2 ▶). The observed structure extends from residue Ser0 (part of the purification tag) to Asp313 in both chains. The loop between residues Ala111 and Thr119 could not be modeled owing to weak electron density. One NADP^+^ molecule and one ethylene glycol molecule were modelled in each chain. The nicotinamide ring of the cofactor is planar; hence, we assume that NADP^+^ is bound to the protein in the oxidized form. The identity of a slightly prolate spheroid of electron density close to the nicotinamide ring of NADP^+^ could not be established and was therefore modelled as ‘unknown atoms’ (UNK). A total of 611 water molecules were located.

## 3. Results and discussion

**Figure 1:**
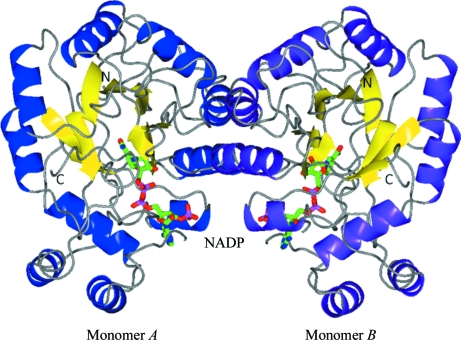
Dimer of aldose reductase from *G. lamblia* (GilaA.01452.aA1). Aldose reductase from *G. lamblia* forms a dimer of two TIM barrels. In this figure the twofold axis runs approximately vertically. The helices of the two monomers are shown as blue and purple ribbons. In each momomer, an NADP molecule is visible at the C-­terminal end of the strands of the β-barrel. This figure was prepared with *CCP*4*mg* (McNicholas *et al.*, 2011 ▶).

*G. lamblia* aldose reductase adopts a TIM-barrel fold with the NADP^+^-binding site located within the eight β-strands of the interior (Fig. 1 ▶). Sequence alignment with *Homo sapiens* aldose reductase shows 44% identity; no structures in the PDB have an identity greater than 50%. The two monomers of *G. lamblia* aldose reductase superimpose with a root-mean-square deviation (r.m.s.d.) of 0.3 Å (on C^α^ atoms); a search of the PDB for structural homologues using *SSM*/*PDBeFold* (Krissinel & Henrick, 2004 ▶) yielded several structures of human aldose reductase with C^α^-atom r.m.s.d.s of 1.0–1.1 Å. When compared with the structure of mammalian aldose reductase, subtle variations in structure are apparent.

There is a distinct structural divergence of the N-termini of *G. lamblia* aldose reductase and the human protein: while the N-­terminal 14 residues of both structures are well ordered, *G. lamblia* aldose reductase lacks the small N-terminal capping β-sheet exhibited by mammalian structures (residues 2–14 of human aldose reductase).

**Figure 2:**
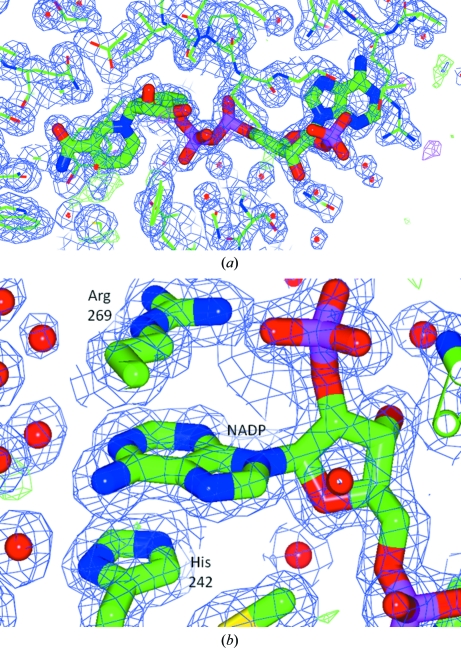
(*a*) NADP is well defined in the structure of *G. lamblia* aldose reductase. (*b*) The adenine ring of NADP stacks hydrophobically with the side chains of His242 and Arg269. While Arg269 is conserved between human and *Giardia* aldose reductase, His242 of *Giardia* aldose reductase is an alanine in the human enzyme. This figure was prepared with *CCP*4*mg* (McNicholas *et al.*, 2011 ▶). In both cases σ_A_-weighted 2|*F*_o_| − |*F*_c_| electron density is shown in blue at 1σ and σ_A_-weighted |*F*_o_| − |*F*_c_| electron density is shown in green and red at ±3σ.

**Figure 3:**
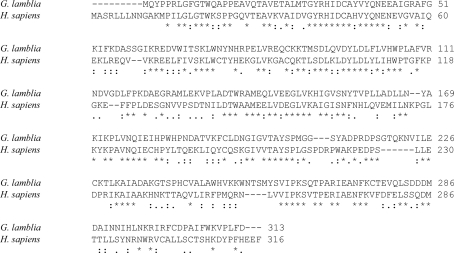
*ClustalW* (Larkin *et al.*, 2007 ▶) comparison of the amino-acid sequences of *H. sapiens* and *G. lamblia* aldose reductase.

Cofactor binding by Arg269 is conserved between the two structures. The side chain of His242 of *G. lamblia* aldose reductase hydrophobically stacks with the adenine of NADP^+^, while this residue is an alanine in the human protein (Fig. 2 ▶). This histidine residue is not found in other aldose reductases of higher organisms and may be unique to *Giardia*. A flexible loop between the strand and helix 7 of the β/α-barrel (dubbed the ‘safety belt’) has previously been reported to sequester solvent from the NADP^+^ cofactor (Wilson *et al.*, 1992 ▶). This flexible feature was not resolved in this structure. Tantalizingly, this region shows divergence between the mammalian and *Giardia* sequences. Sequence comparison with human aldose reductase by *ClustalW* (Larkin *et al.*, 2007 ▶) analysis (Fig. 3 ▶) indicates that phenylalanine residues at positions 109 and 118 are conserved participants in forming the hydrophobic substrate-specificity pocket. Other residues identified as components of the mammalian specificity pocket (Klebe *et al.*, 2004 ▶; Harrison *et al.*, 1994 ▶; Sotriffer *et al.*, 2004 ▶; Urzhumtsev *et al.*, 1997 ▶) are divergent (Table 3 ▶). The residues of the anion-binding pocket and the active site appear to be completely conserved (Table 3 ▶) and their orientation is conserved in the three-dimensional structure. The residues flanking the C-terminal mobile region appear to be conservative substitutions, while the interior residues of this short region show no conservation (Table 3 ▶).

**Figure 4:**
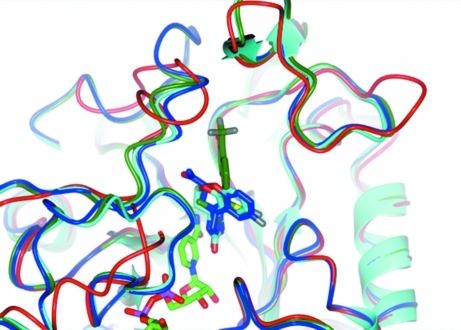
Inhibitor-binding pocket of *G. lamblia* aldose reductase in comparison with ligand-bound human and porcine aldose reductases. *G. lamblia* aldose reductase (red model) superimposes with small r.m.s.d.s with human aldose reductase in complex with fidarestat (PDB entry 1ef3, dark blue) and porcine aldose reductase in complex with tolrestat (PDB entry 1ah3, green) and sorbinil (PDB entry 1ah0, cyan). Despite the overall structural homology, the C-terminal loop, which interacts with the ligand, shows a distinctly different trace. For clarity, the C atoms of the inhibitors follow the same color scheme as the corresponding ribbon. The NADP molecule is shown only for *G. lamblia*, with light green C atoms. This figure was prepared with *CCP*4*mg* (McNicholas *et al.*, 2011 ▶).

The structure of the *G. lamblia* aldose reductase was superimposed with ligand-bound aldose reductases such as human aldose reductase in complex with fidarestat (PDB entry 1ef3; Oka *et al.*, 2010 ▶) and porcine aldose reductase in complex with tolrestat (PDB entry 1ah3; Urzhumtsev *et al.*, 1997 ▶) or sorbinil (PDB entry 1ah0; Urzhumtsev *et al.*, 1997 ▶). All three inhibitors bind in close proximity to the nicotin­amide group of NADP (Fig. 4 ▶). The protein models superimpose well overall, with r.m.s.d.s of 1.06 Å (1ef3), 1.03 Å (1ah3) and 1.13 Å (1ah0). Despite high overall structural homology, there is a distinct difference in structure, separate from the mobile regions defined earlier, between the *G. lamblia* and mammalian enzymes close to the inhibitor-binding site. The C-terminal loop formed by residues Pro303–Leu311 diverges significantly from the structure of the corresponding loop in the human and porcine structures. In the human and porcine structures this loop is much more conserved and interacts with the inhibitors. In addition, structural rearrangements would be necessary for *G. lamblia* aldose reductase to harbor any of these three inhibitors. This structural diversity of the ligand-binding pocket in turn provides opportunities for the design of drugs that are specific for *G. lamblia* aldose reductase.

## 4. Conclusion

The structure of *G. lamblia* aldose reductase in complex with NADP^+^ was solved to a resolution of 1.75 Å. Residues in the active site and anion-binding pocket show conservation in sequence and structure with mammalian structures, while the nonphenylalanine residues involved in forming the specificity pocket show divergence. This structure did not resolve a flexible loop involved in solvent sequestration of NADP^+^. A conserved arginine residue is involved in cofactor binding together with an apparently *Giardia*-specific histidine residue.

## Supplementary Material

PDB reference: aldose reductase, 3krb

## Figures and Tables

**Table 1:** Data-collection statistics Values in parentheses are for the highest resolution shell.

Beamline	ALS 5.0.1
Wavelength (Å)	0.9774
Space group	*C*2
Unit-cell parameters (Å, °)	*a* = 196.77, *b* = 66.09, *c* = 56.29, β = 92.26
Resolution range (Å)	50–1.75 (1.80–1.75)
Mean *I*/σ(*I*)	17.0 (2.5)
*R*_merge_†	0.073 (0.558)
Completeness (%)	99.9 (99.8)
Multiplicity	4.6 (4.1)
No. of unique reflections	72824 (5374)

† *R*_merge_ = 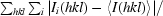 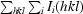.

**Table 2:** Refinement and model statistics

Beamline	ALS 5.0.1
Resolution range (Å)	50–1.75
*R*_cryst_†	0.144
*R*_free_†	0.173
R.m.s.d. bonds (Å)	0.016
R.m.s.d. angles (°)	1.56
Protein atoms	4908
Nonprotein atoms	767
Mean *B* factor (Å^2^)	16.7
Residues in favoured region	580 (97.2%)
Residues in allowed region	17 (2.9%)
Residues in disallowed region	0 (0%)
*MolProbity*‡ score (percentile)	1.13 (99th)
PDB code	3krb

† *R*_cryst_ = 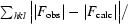 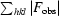. The free *R* factor was calculated using 5% of the reflections omitted from the refinement (Winn *et al.*, 2011 ▶).

‡ Chen *et al.* (2010 ▶).

**Table 3:** Conservation of residues involved in substrate binding

	*H. sapiens*	*G. lamblia*
Specificity pocket	Thr114	Leu107
	Cys304	Ala304
	Tyr310	Pro310
Anion-binding pocket	Trp21	Trp12
	Val48	Val39
	Tyr49	Tyr40
	His111	His104
	Trp112	Trp105
	Lys78	Lys71
	Asp44	Asp35
C-terminal mobile region	Val298	Ile297
	Cys304	Ala304
	Ala299	Phe299
	Leu300	Cys301
